# Complication rates as a trauma care performance indicator: a systematic review

**DOI:** 10.1186/cc11680

**Published:** 2012-10-16

**Authors:** Lynne Moore, Henry Thomas Stelfox, Alexis F Turgeon

**Affiliations:** 1 Department of Social and Preventive Medicine, Université Laval, 2325 rue de l'Université, Québec City, Québec, Canada G1V 0A6; 2 Department of Critical Care Medicine, Medicine and Community Health Sciences (HTS), Institute for Public Health, University of Calgary, 2500 University Dr. NW, Calgary, Alberta, Canada T2N 1N4; 3 Axe traumatologie-urgence-soins intensifs, Centre de Recherche FRQ-S du CHA-Hôpital Enfant-Jésus, 1401 18e rue, Quebec City, Québec, Canada G1J 1Z4; 4 Department of Anesthesiology, Division of Critical Care Medicine, Université Laval, 2325 rue de l'Université, Québec City, Québec, Canada G1V 0A6

## Abstract

**Introduction:**

Information on complication rates is essential to trauma quality improvement efforts. However, it is unclear which complications are the most clinically relevant. The objective of this study was to evaluate whether there is consensus on the complications that should be used to evaluate the performance of acute care trauma hospitals.

**Methods:**

We searched the Medline, EMBASE, Cochrane Central, CINAHL, BIOSIS, TRIP and ProQuest databases and included studies using at least one nonfatal outcome to evaluate the performance of acute care trauma hospitals. Data were extracted in duplicate using a piloted electronic data abstraction form. Consensus was considered to be reached if a specific complication was used in ≥ 70% of studies (strong recommendation) or in ≥ 50% of studies (weak recommendation).

**Results:**

Of 14,521 citations identified, 22 were eligible for inclusion. We observed important heterogeneity in the complications used to evaluate trauma care. Seventy-nine specific complications were identified but none were used in ≥ 70% of studies and only three (pulmonary embolism, deep vein thrombosis, and pneumonia) were used in ≥ 50% of studies. Only one study provided evidence for the clinical relevance of complications used and only five studies (23%) were considered of high methodological quality.

**Conclusion:**

Based on the results of this review, we can make a weak recommendation on three complications that should be used to evaluate acute care trauma hospitals; pulmonary embolism, deep vein thrombosis, and pneumonia. However, considering the observed disparity in definitions, the lack of clinical justification for the complications used, and the low methodological quality of studies, further research is needed to develop a valid and reliable performance indicator based on complications that can be used to improve the quality and efficiency of trauma care.

## Introduction

Complications following admission for traumatic injury are common and have been shown to increase hospital mortality, length of stay, and costs [1-5]. These complications have been associated with a negative impact on long-term functional capacity and quality of life [6]. Many complications are potentially avoidable and quality improvement strategies aimed at reducing them have been shown to have a positive impact on patient outcome and resource utilization [7,8].

Complications have been identified as a priority for the development of trauma performance indicators [9,10]. Furthermore, US Trauma Quality Improvement Program members have made efforts to track information on complications following trauma using the National Trauma Data Bank according to the National Surgical Quality Improvement program [11,12]. However, despite the widespread availability of routinely collected data on hospital complications, a performance indicator based on complications has yet to be validated specifically in the context of acute trauma care [9,10]. Considering the potential positive and negative consequences of healthcare performance evaluation [13], such a performance indicator should be based on clinically relevant complications identified using standardized definitions [14] and robust methodology including adequate risk adjustment [15].

A recent systematic review synthesized studies evaluating performance indicators in trauma care [9]. However, information specific to the assessment of complications in the context of acute trauma care is limited. The objective of this study was to evaluate whether there is consensus on the complications that should be used to evaluate the performance of acute care trauma hospitals.

## Materials and methods

We designed a systematic review of cohort studies evaluating the performance of acute care hospitals for the treatment of general trauma populations using information on complications. This systematic review was conducted following recommendations from the Cochrane handbook for systematic reviews [16] and in compliance with the Preferred Reporting Items for Systematic Reviews and Meta-Analyses (PRISMA) statement [17]. The study was approved by our institutional research ethics committee. The present systematic review complements a review published by one of the authors (HTS) in 2011. The latter review was designed to document evidence of the reliability and validity of trauma quality indicators at large, whereas the present study is designed to evaluate whether there is consensus on the complications used to evaluate trauma care quality.

### Search strategy

We searched the Medline, EMBASE, Cochrane Library, CINAHL, BIOSIS, TRIP and ProQuest databases for peer-reviewed articles and postgraduate academic publications. The websites of the following trauma associations were also searched: American College of Surgeons, American Association for the Surgery of Trauma, Eastern Association for the Surgery of Trauma, American Trauma Society, British Trauma Society, Trauma Association of Canada, Australasian Trauma Society, Western Trauma Association, Trauma.org, Society of Trauma Nurses, and International Trauma Anaesthesia and Critical Care Society. Our initial systematic search strategy was based on nonfatal outcomes at large. We then selected studies based on complications for this systematic review. The search strategy was designed for Medline and EMBASE using keywords and MeSH (Medline) or Emtree (EMBASE) for three groups of terms: trauma, performance, and nonfatal outcomes (see Additional file 1). Keywords were elaborated by a group of experts with methodological and clinical expertise who are co-investigators and collaborators on the project. The systematic search was limited to human studies from high-income countries conducted from the earliest available date of the search engine to 1 July 2011. The bibliographies for all identified studies and reviews were searched manually to identify additional relevant publications. No publication type, year, or language restriction was used.

### Study selection

Both prospective and retrospective cohort studies evaluating the performance of acute care hospitals for the treatment of global trauma populations using at least one medical complication were considered eligible. A complication was defined as an additional problem that arises following a procedure, treatment, or illness and is secondary to it [18].

Duplicates were identified and sorted by two independent reviewers with methodological and content expertise (LM and HTS) using EndNote software version X4 (2010; Thomson Reuters, New York, NY, USA). These two reviewers independently evaluated citations identified for eligibility by screening titles, abstracts, and full publications. Disagreement on study eligibility was resolved by consensus, and a third reviewer (AFT) was involved when required. Inter-rater agreement was evaluated with kappa statistics on study eligibility. Articles written in a language other than English were translated.

### Data abstraction

The same two reviewers independently extracted data using a standardized data abstraction form, which was piloted on a sample of five representative studies. The data abstraction form was designed to capture information on the study setting and design, complications, and methodological quality. The latter was evaluated using elements selected from the following sources: a tool proposed to evaluate the quality of complication-reporting in the surgical literature [14], the Strengthening the Reporting of Observational Studies in Epidemiology statement [19], the Cochrane risk of bias tool [20], and the Downs and Black tool [21]. The following 10 methodological quality criteria were thus selected by the project steering committee based on the consideration that they are important for the validity and reliability of performance evaluations: definitions of complications provided; justification for the choice of specific complications provided; duration of follow-up indicated; number and percentage of specific complications indicated; severity of complications considered (for example, major versus minor complications); risk adjustment used; data quality assurance efforts reported; adequate treatment of missing data; estimates of variation given; and adequate sample size.

Adequate treatment of missing data implied that the absence/presence and proportion of missing data were reported, and if > 10% of subjects had missing data then imputation techniques that take account of the uncertainty of missing data values (for example, multiple imputation/maximum expectation) or sensitivity analysis were used [22]. Sample size was considered adequate if at least 100 patients per hospital were available for analysis; if not, analysis strategies designed for low-volume centers (for example, shrinkage techniques) were used [15]. Disagreement on abstracted data between reviewers was resolved by consensus or, if necessary, consultation with a third reviewer (AFT).

### Analysis

A classification system for complications following traumatic injury proposed elsewhere [23] was used to categorize specific complications identified in this review. Complications were thus grouped into the following 10 categories: pulmonary, cardiovascular, gastrointestinal, hepatic/biliary, hematologic, infection, genitourinary/renal, musculoskeletal/integumentary, neurologic, vascular, psychiatric, and other. According to Cochrane GRADE criteria for clinical recommendations [24], consensus was considered to be reached for a particular complication if at least 70% (strong recommendation) or 50% (weak recommendation) of studies used that complication to evaluate trauma care. Sensitivity analyses were performed to see whether greater consensus was achieved in more recent studies (later than 2005). Studies were considered to be of high methodology quality if at least seven out of 10 quality criteria were respected [25].

## Results

### Search results

We identified 14,521 citations, of which 112 were selected for full text review after screening of titles and abstracts (Table 1). A total of 22 studies met the inclusion criteria [26-47]. We had excellent inter-rater agreement on the selection of eligible studies with a kappa statistic of 0.89 (95% confidence interval = 0.79 to 0.99).

**Table 1:** Description of included studies

Author, year (reference)	Setting	Study design	Period	Patients (*n*)	Centers (*n*)	Age (years)	Mechanism (% blunt)	Gender (% male)	ISS
Ang and colleagues, 2009 [27]	NSCOT, USA	Prospective	2001 to 2002	5,043	69	29% ≥ 65	88	62	74% ≥ 16
Calderale and colleagues, 2008 [28]	Level I TC, Italy/Romania	Retrospective	2002	182	2	Mean = 43	74% MVC	80	Mean = 29
Chang and colleagues, 2008 [29]	NIS, USA	Retrospective	2000 to 2004	1,350,229	NA	Mean = 60	NR	48	Median = 8
Claridge and colleagues, 2001 [30]	Level I TC, USA	Retrospective	1994 to 1999	917	1	Mean = 41	88	67	Mean = 14
Cohen and colleagues, 1999 [31]	Level I TC, USA	Retrospective	1995 to 1997	1,025	1	Mean = 43	75	66	Mean = 9
Curtis and colleagues, 2002 [32]	TC, Australia	Retrospective	2000 to 2001	475	1	NR	NR	NR	NR
Davis and colleagues, 2008 [33]	Level I TC, USA	Retrospective	2005 to 2006	1,058	1	NR	85	NR	Mean = 13
DiRusso and colleagues, 2001 [34]	Level I TC, USA	Retrospective	1994 and 1998	2,774	1	Mean = 36	94	57	Mean = 11
Glance and colleagues, 2011 [35]	NTDB, USA	Retrospective	2007	54,713	42	Median = 37	91	67	NR
Haas and colleagues, 2011 [36]	NTDB, USA	Retrospective	2007	76,048	115	Mean = 45	90	69	49% > 15
Haut and colleagues, 2007 [37]	Level I TC, USA	Retrospective	1995 to 2005	7,559	1	Mean = 33	52	80	Mean = 10
Highstead and colleagues, 2009 [38]	Level I TC, USA	Retrospective	1998 to 2007	7,593	1	Mean = 36	78	76	Mean = 8
Hinsdale and colleagues, 1998 [39]	TC, USA	Retrospective	1993 to 1996	6,992	1	NR	NR	NA	NR
Hoyt and colleagues, 2003 [26]	Level I TC, USA	Retrospective	1990 to 2001	13,382	1	NR	71	78	Mean = 13
Huseynova and colleagues, 2009 [40]	NTDB, USA	Retrospective	2006	22,421	30	Mean = 45	90	68	Mean = 17
Jacobs and colleagues, 2009 [41]	Level I TC, USA	Retrospective	2006	1,959	1	Mean = 37	82	72	Mean = 15
Pierce and colleagues, 2008 [42]	NTDB, USA	Retrospective	2001 to 2005	578,252	147	NR	NR	NA	NR
Piontek and colleagues, 2003 [43]	Level I TC, USA	Retrospective	1993 to 2001	7,811	1	NR	97	NR	NR
Podnos and colleagues, 1998 [44]	Level I TC, USA	Retrospective	1996	1,427	1	NR	NR	73	NR
Roettger and colleagues, 2005 [45]	Level I TC, USA	Retrospective	2001 to 2004	1,391	1	Mean = 37	87	66	Mean = 11
Rotondo and colleagues, 2009 [46]	Level I TC, USA	Retrospective	1994 to 2005	18,644	1	Mean = 41	88	63	Mean = 11
Schuerer and colleagues, 2005 [47]	Level I TC, USA	Prospective	2002 to 2003	2,531	1	Mean = 39	NR	NR	Median = 9

ISS, Injury Severity Score; MVC, motor vehicle collision; NA, not applicable; NIS, National Inpatient Survey; NR, not reported; NSCOT, National Study on Costs and Outcomes of Trauma; NTDB, National Trauma Data Bank; TC, trauma center;

### Study characteristics

Of 22 selected studies, 20 (91%) were conducted in the USA [26,27,29-31,33-47], one in Europe [28], and one in Australia [32]. The studies spanned data collected from 1990 [26] through 2007 [35,36,38]. The majority of studies were based on a single trauma center (*n *= 15) [26,30-34,37-39,41,43-47], six were based on national (USA) trauma data [27,29,35,36,40,42], and one was based on the comparison of two trauma centers [28]. All but two studies [27,47] were retrospective cohort studies. The mean Injury Severity Score varied between 8 and 29 [26,28,30,31,33,34,37,38,40,41,45,46]. The proportion of blunt trauma varied between 52% [37] and 97% [43]. Six studies were restricted to adult trauma patients [27,29,32,36,37,40], while none of the other studies mentioned inclusion or exclusion criteria based on age.

### Complications

Four studies (18%) did not provide any information on the specific complications used to evaluate performance [31,32,38,44]. Among the other studies, we observed important heterogeneity in the complications used (Table 2). A total of 79 different complications were identified. None of the complications were used in more than 70% of studies (consistent with a strong recommendation) but the following complications were used in at least 50% of studies (weak recommendation): pulmonary embolism (*n *= 12), deep vein thrombosis (*n *= 12), and pneumonia (*n *= 11). Other commonly evaluated complications were sepsis (*n *= 8), urinary tract infection (*n *= 8), renal failure (*n *= 7), myocardial infarction (*n *= 7), wound infection (*n *= 7), decubitus ulcer (*n *= 6), respiratory failure (*n *= 6), and acute respiratory distress syndrome (*n *= 6).

**Table 2:** Identification of acute care medical complications

	Reference																	
	
	[27]	[28]	[29]	[30]	[33]	[34]	[35]	[36]	[37]	[39]	[26]	[40]	[41]	[42]	[43]	[45]	[46]	[47]
**Pulmonary**																		
Pulmonary embolism^a^	X	X	X			X	X		X	X	X	X				X	X	X
Pneumonia^a^	X			X	X	X	X	X		X	X		X		X	X		
ARDS							X	X		X	X		X			X		
Respiratory failure						X				X	X		X			X	X	
Hemothorax/pneumothorax			X							X	X					X		
Aspiration/pneumonia										X	X					X		
Empyema										X	X					X		
Pleural effusion										X	X		X					
Abscess										X	X							
Atelectasis										X	X							
Fat embolus										X	X							
Pulmonary edema										X	X							
Respiratory failure/distress										X	X							
**Infections**																		
Sepsis	X				X	X	X	X		X	X		X					
Wound	X			X						X	X		X		X	X		
Sepsis-like syndrome										X	X		X			X		
Catheter-related (line infection)				X						X	X							
Disseminated fungal										X	X					X		
Intra abdominal										X	X					X		
Sinusitis										X	X					X		
Cellulitis/traumatic										X	X							
Necrotizing fascitis										X	X							
Yeast										X	X							
Bloodstream				X														
Postoperative															X			
**Vascular**																		
Deep vein thrombosis^a^	X	X	X						X	X	X	X		X	X	X	X	X
Anastomosis hemorrhage										X	X							
Embolus (nonpulmonary)										X	X							
Gangrene										X	X							
Graft infection										X	X							
Thrombosis										X	X							
**Genitourinary/renal**																		
Urinary tract infection	X			X	X	X				X	X		X			X		
Renal failure						X	X	X		X	X		X			X		
Urethral injury										X	X							
**Cardiovascular**																		
Myocardial infarction							X	X		X	X				X	X	X	
Arrhythmia	X									X	X		X			X		
Cardiac arrest	X							X		X	X					X		
Shock										X	X				X	X		
Congestive heart failure										X	X					X		
Cardiogenic shock										X	X							
Pericardial effusion or tamponade										X	X							
Pericarditis										X	X							
**Neurologic**																		
Stroke							X	X		X	X						X	
Progression of original neurologic insult										X	X					X		
Alcohol withdrawal										X	X							
Anoxic encephalopathy										X	X							
Diabetes insipidus										X	X							
Meningitis										X	X							
Neuropraxia (iatrogenic)										X	X							
Nonoperative SDH/EDH										X	X							
Seizure in hosp										X	X							
SIADH										X	X							
Ventriculitis										X	X							
**Gastrointestinal**																		
Evisceration/dehiscence										X	X					X		
Gastro-intestinal fistula										X	X					X		
Peritonitis				X						X	X							
Small bowel obstruction										X	X					X		
Abdominal compartment syndrome							X									X		
Anastomic leak										X	X							
Bowel injury (iatrogenic)										X	X							
Hemorrhage										X	X							
Ileus										X	X							
Ulcer-duodenal/gastric										X	X							
Gastrointestinal bleeding/stress ulceration																X		
Enterotomy																		
**Hematologic**																		
Coagulopathy	X						X			X	X					X		
Transfusion complication			X							X	X							
**Musculoskeletal/integumentary**																		
Decubitus ulcer (skin breakdown)		X	X							X	X		X			X		
Extremity compartment syndrome										X	X					X		
Nonunion										X	X							
Osteomyelitis										X	X							
**Hepatic/biliary**																		
Hepatic (liver) failure										X	X					X		
Pancreatitis										X	X					X		
Acalculous cholecystitis										X	X							
Hepatitis										X	X							
Pancreatic fistula										X	X							
Splenic injury										X	X							
**Psychiatric**																		
Psychiatric										X	X							
**Other**																		
Hypothermia																X		

ARDS, acute respiratory distress syndrome; EDH, epidural hematoma; SDH, subdural hematoma; SIADH, syndrome of inappropriate diuretic hormone secretion. ^a^Complications used in at least 50% of studies (consistent with weak clinical recommendations)

### Sensitivity analyses

Among 11 studies published later than 2005 [27-29,33,35-37,40-42,46], we observed similar results; pulmonary embolism, deep vein thrombosis, and pneumonia were used in more than 50% of studies.

### Methodological quality

Only five studies (23%) were considered of high methodological quality with at least seven out of 10 quality criteria respected (Table 3). Eight studies gave no definition of the complications used to evaluate performance (Table 3) [31,32,34,37,38,41,44,46]. Six studies gave medical (*n *= 3) [27,35,47] or diagnostic code (*n *= 4) [30,33,40,42] definitions of specific complications, whereas seven studies provided a reference for the definition of selected complications [26,28,29,36,39,43,45]. Thirteen studies (59%) gave no justification for their choice of complications [27,30-35,37,38,41,44,46,47]. Only one study justified the clinical relevance of included complications [36]; the authors selected eight complications reported to have the highest attributable mortality in a trauma population [1]. Three studies used certain complications recommended for quality improvement activities by the US Agency for Health Care Research and Quality [29,40,42]. Two studies used certain complications identified by the American College of Surgeons Committee on Trauma [28,45]. Two studies used complications observed in an independent trauma population [26,39], and one study [43] was based on complications used to evaluate quality in a general admission population [15].

**Table 3:** Methodological quality of included studies

Study	Definitions of complications	Justification for choice of complications	Duration of follow-up	Number and percentage of specific complications	Complication severity considered	Risk adjustment	Data quality assurance efforts	Adequate treatment missing data	Estimates of variation	Adequate sample size
Ang and colleagues [27]^a^	X			X		X	X	X	X	X
Calderale and colleagues [28]	X	X								X
Chang and colleagues [29]	X	X				X		X	X	X
Claridge and colleagues [30]	X									X
Cohen and colleagues [31]										X
Curtis and colleagues [32]										X
Davis and colleagues [33]	X								X	X
DiRusso and colleagues [34]					X					X
Glance and colleagues [35]^a^	X		X		X	X		X	X	X
Haas and colleagues [36]^a^	X	X	X	X	X	X		X		X
Haut and colleagues [37]		X		X						
Highstead and colleagues [38]				X						X
Hinsdale and colleagues [39]	X	X							X	X
Hoyt and colleagues [26]	X	X	X							
Huseynova and colleagues [40]^a^	X	X	X	X		X			X	X
Jacobs and colleagues [41]				X					X	X
Pierce and colleagues [42]^a^	X	X	X	X		X			X	X
Piontek and colleagues [43]	X	X				X			X	X
Podnos and colleagues [44]			X	X					X	X
Roettger and colleagues [45]	X	X		X						X
Rotondo and colleagues [46]				X					X	X
Schuerer and colleagues [47]	X			X						

^a^Study considered to be of high methodological quality with at least seven out of 10 criteria respected

Among the 22 studies included, six (15%) indicated the duration of follow-up [26,35,36,40,42,44], which was always until hospital discharge. The number and proportion of each specific complication were given in 11 studies [27,36-38,40-42,44-47]. The severity of complications (that is, major versus minor) was specified in three studies [34-36]. Nearly all studies had adequate sample size to evaluate performance, and 50% presented estimates of variation (that is, standard errors or confidence intervals). However, less than one-half of the studies used risk adjustment, one study reported using data quality assurance [27], and four studies adequately addressed the problem of missing data [27,29,35,36].

## Discussion

In this systematic review, we identified 79 specific complications that have been used to evaluate acute care trauma hospitals. None of these complications were consistent with a strong clinical recommendation but pulmonary embolism, deep vein thrombosis, and pneumonia were used in over 50% of studies and were therefore consistent with a weak clinical recommendation. Studies included in our review rarely justified the choice of complications included, and they generally had low methodological quality.

Reducing hospital complications is one of the keys to reducing morbidity, mortality, and resource utilization following trauma [1,3-5]. Evaluating complications is therefore essential to improve the quality of care for patients admitted to acute care institutions for traumatic injuries. However, valid and reliable performance indicators are dependent on standardized definitions and rigorous methodological quality [15,48,49].

Standardized definitions should include a consensus on which complications should be used, how they should be defined, and the timing of evaluation [15,48,49]. First, the results of this study show that there is currently a lack of consensus on which complications should be used to evaluate trauma care; no single complication was used in more than 70% of studies, only two out of 22 studies shared the same list of specific complications [26,39], less than one-half gave justifications for the complications they used to evaluate care, and only one study demonstrated the clinical relevance of included complications [36]. This finding is supported by a previous study in a surgical population [14]. Second, less than one-half of studies provided definitions of specific complications, a problem noted by others [14]. Third, outcomes should be evaluated over a fixed period of time and should include early post-discharge data (for example, complications within the first 28 days of injury) [15]. Indeed, research has shown that major complications do occur after discharge from acute care [50]. In our systematic review, all studies evaluated complications from admission to discharge, which may unfairly advantage hospitals discharging patients early. Assessing complications after discharge is challenging but trauma registries may be linked to hospital discharge datasets to obtain information on complications that have led to hospital readmission.

Rigorous methodological quality includes, among other considerations, data quality and appropriate risk adjustment [15,48]. In this review, data quality assurance was mentioned in only one study [27] and less than one-third of studies used any type of risk adjustment. The problem of data quality or missing data in retrospective evaluations of hospital complications, underlined in our study, has been raised in previous studies [12,51,52]. Authors note that complications which are not recorded either due to differential surveillance or because of poor data quality are assumed to be absent, which leads to an underestimation for the incidence of hospital complications. Robust risk adjustment is essential to valid performance comparisons because of the heterogeneous case mix across trauma centers [15]. Indeed, the risk of complications has been reported to vary according to age, gender, injury severity, and comorbidities [18,53]. Distinguishing preventable from nonpreventable complications is also an important challenge in quality evaluations [48], that was partially addressed by three studies included in this review [26,29,39].

### Potential limitations

The results of this systematic review should be interpreted in light of possible limitations. First, despite the exhaustive nature of our search strategy and very good inter-rater agreement on study eligibility, some studies may have been missed. For feasibility reasons, we restricted our website search to major trauma organizations in North America, Europe, and Australasia. By doing so, we may have missed some local studies based on regional trauma organizations. In the event that studies were missed, we may have overlooked some specific complications. Second, missing information in study reports meant that we did not have information on some study characteristics, definitions, and methodological quality criteria. For the latter, lack of information was assumed to mean that they did not meet the criteria and may have led to an underestimation of methodological quality. However, adequate reporting of important information is a reflection of study quality [19]. Third, complications that have been used for performance evaluation of trauma care may not necessarily represent those that researchers and stakeholders consider to be the most clinically relevant. For example, psychiatric complications were only evaluated in two studies and we did not identify any specific psychiatric complications - such as delirium, which is frequent in trauma populations [54]. In addition, the complications identified in this study are likely to have been heavily influenced by data availability, considering that most were based on retrospectively collected administrative or registry data. This influence highlights the importance of using a consensus-based procedure as well as information from a literature review to identify which complications should be used to evaluate trauma care.

## Conclusion

According to the GRADE criteria, we can make a weak recommendation on three complications that should be used to evaluate the performance of acute care trauma hospitals: pulmonary embolism, deep vein thrombosis, and pneumonia. However, considering the heterogeneity of definitions used, the lack of clinical justification for the choice of specific complications, and the low methodological quality of included studies, further research is needed to develop a valid and reliable performance indicator based on complications that can be used to improve the quality and efficiency of trauma care.

## Key messages

• Evidence in the literature is sufficient to make a weak recommendation on three complications that should be used to evaluate trauma center care; pulmonary embolism, deep vein thrombosis, and pneumonia.

• Definitions of complications are heterogeneous.

• Studies that use complications to evaluate trauma center care are generally of low methodological quality.

• The choice of complications is rarely justified by clinical criteria.

• Further research is needed to develop a valid and reliable performance indicator based on complications that can be used to improve the quality and efficiency of trauma care.

## Abbreviations

GRADE: Grading the Quality of Evidence and Strength of Recommendations in ATS Guidelines and Recommendations.

## Competing interests

The authors declare that they have no competing interests.

## Authors' contributions

LM, HTS, and AFT made substantial contributions to conception and design, acquisition of data, as well as the analysis and interpretation of data. LM, HTS, and AFT were involved in drafting the manuscript or revising it critically for important intellectual content. LM, HTS, and AFT gave final approval of the version to be published. Each author participated sufficiently in the work to take public responsibility for appropriate portions of the content. All authors read and approved the final manuscript.

## Supplementary Material

Additional file 1 **Diagram presenting MeSH and keywords used in the Medline search strategy**. Click here for file
