# Supplementary material for: Complication rates as a trauma care performance indicator: a systematic review
Source: Crit Care. 2012 Oct 16;16(5):R195. doi: 10.1186/cc11680 (PMC3682297; doi:10.1186/cc11680)
Supplement: Additional file 1 — Diagram presenting MeSH and keywords used in the Medline search strategy. [file cc11680-S1.DOCX]

**Medline search strategy**

1. trauma$.mp. or injur$.tw.

2. exp "Wounds and Injuries"/

3. 1 or 2

4. exp Quality Indicators, Health Care/ or Quality Assurance, Health Care/ or Benchmarking/ or Total Quality Management/ or "health care quality, access, and evaluation"/

5. ((quality or process) adj2 (measure$ or indicator? Or compar*)).tw. or (quality adj2 (assurance? Or evaluation$ or control$ or assessment$)).tw.

6. (best practi?e?).tw.

7. benchmark*.tw.

8. (performance$ adj2 (improvement? Or evaluation? or measur$ Or assessment? Or compar*)).tw.

9. (audit filter?).tw.

10. ((trauma* or surg$) adj audit?).tw.

11. "risk adjusted scor$".tw.

12. health service* evaluation*.tw.

13. 4 or 5 or 6 or 7 or 8 or 9 or 10 or 11 or 12

14. ((treatment? or assessment) adj2 outcome$).tw.

15. "outcome assessment (health care)"/ or "process assessment (health care)"/

16. health status.tw.

17. medical error*.tw. OR medical mistake*.tw. OR exp Medical Errors/

18. safety.tw.

19. morbidit*.mp. OR exp Morbidity/

20. adverse event$.tw.

21. complication$.mp.

22. myocardial infarct*.tw. OR MI.tw. OR exp Myocardial Infarction/

23. cardiac arrest.tw. OR heart arrest.tw. OR cardiopulmonary arrest.tw. OR exp Heart Arrest/

24. stroke*.tw. OR exp Stroke/

25. brain vascular accident*.tw. OR cerebrovascular accident*.tw. OR exp Cerebrovascular Disorders/ OR exp Brain Ischemia/ OR exp "Intracranial Embolism and Thrombosis"/ OR exp Ischemic Attack, Transient/

26. cerebrovascular disorder*.tw. OR brain Ischemia.tw. OR intracranial embolism thrombosis.tw. OR transient ischemic attack*.tw. OR cva.tw. OR cvas.tw.

27. pulmonary embolism.tw. OR PE.tw. OR pulmonary thromboembolism*.tw. OR exp Pulmonary Embolism/

28. deep vein thrombosis.tw. OR phlebothrombos*.tw. OR deep venous thrombosis.tw. OR venous thromboembolism.tw. OR thrombophlebitis.tw. OR venous thrombosis.tw. OR DVT.tw. OR exp Venous Thrombosis/ OR exp Thrombophlebitis/ OR exp Venous Thromboembolism/ OR exp Pulmonary Embolism/ OR exp Thromboembolism/

29. acute renal insufficienc*.tw. OR acute kidney failure.tw. OR acute kidney insufficienc*.tw. OR acute renal failure.tw. OR exp Acute Kidney Injury/

30. liver failure.tw. OR hepatic failure.tw. OR shock liver.tw. OR exp Liver Failure/

31. pneumonia*.tw. OR aspiration pneumonia*.tw. OR exp Pneumonia/

32. respiratory failure.tw. OR respiratory insufficienc*.tw. OR exp Respiratory Insufficiency/

33. acute respiratory distress syndrome.tw. OR ARDS.tw. OR exp Respiratory Distress Syndrome, Adult/

34. septicemia*.tw. OR sepsis.tw. OR septic shock.tw. OR exp Sepsis/

35. bacteremia*.tw. OR blood stream infection$.tw.

36. wound infection$.tw. OR exp Wound Infection/

37. iatrogenic pneumothorax.tw.

38. hypothermia.tw. OR exp Hypothermia/

39. shock.tw. OR exp Shock/

40. compartmental syndrome*.tw. OR compartment syndrome*.tw. OR exp Compartment Syndromes/

41. delirium.tw. OR exp Delirium/

42. pseudomembranous colitis.tw. OR ((c-difficile or c difficile or Clostridium difficile) adj2 colitis).tw. OR exp Enterocolitis, Pseudomembranous/ OR exp Clostridium Infections/

43. urinary tract infection$.tw. OR exp Urinary Tract Infections/

44. Coagulopath*.tw. OR blood coagulation disorder*.tw. OR exp Blood Coagulation Disorders/

45. decubitus ulcer$.tw. OR skin breakdown.tw. OR skin ulcer*.tw. OR pressure ulcer*.tw. OR pressure sore*.tw. OR bed sore*.tw. OR exp Pressure Ulcer/

46. renal failure*.tw. OR renal insufficienc*.tw. OR kidney failure*.tw. OR kidney insufficienc*.tw. OR exp Renal Insufficiency/

47. exp Cross Infection/ OR exp Wound Infection/ OR exp Surgical Wound Infection/

48. (resistan$ and infection$).tw.

49. MRSA.tw. OR methicilin resistant staphylococcus aureus.tw. OR exp Methicillin-Resistant Staphylococcus aureus/

50. VRE.tw. OR vancomycin resistant enterococcus.tw. OR exp Vancomycin Resistance/

51. resist* c-difficile.tw. OR resist* acinetobacter.tw. OR resist* enterobacter.tw. OR resist* enterobacteria.tw. OR resist* pseudomonas aeruginosa.tw. OR resist* escherichia coli.tw. OR Glycopeptide* resistant enterococci.tw.

52. exp Drug Resistance, Microbial/ OR exp Drug Resistance, Bacterial/ OR exp Drug Resistance, Multiple, Bacterial/

53. length of stay.tw. OR stay length*.tw. OR length of hospital stay.tw. OR hospital stay.tw. OR stay in hospital.tw. OR inpatient stay.tw. OR days in hospital.tw. OR duration of stay.tw. OR exp "Length of Stay"/

54. icu length of stay.tw. OR length of stay in icu.tw. OR duration of ICU stay.tw. OR intensive care unit length of stay.tw.

55. (duration adj2 (mechanical ventilation)).tw.

56. return to work.tw. OR return to employment.tw. OR exp Rehabilitation, Vocational/

57. return to usual activit$.tw. OR return to pre-injury levels of activit$.tw. OR return to major activit$.tw. OR return to usual major activit$.tw. OR return to normal daily activit$.tw. OR return to independent living.tw.

58. exp "Activities of Daily Living"/ or exp Self Care/

59. (level adj2 function$).tw. OR recovery.tw. OR exp "Recovery of Function"/

60. quality of life.tw. OR quality-Adjusted Life Years.tw. OR QUALY.tw. OR exp Quality-Adjusted Life Years/

61. functional capacity index.tw. OR FCI.tw. OR functional independence measure.tw. OR FIM.tw.

62. exp Disability Evaluation/

63. Glasgow Outcome Scale.tw. OR GOS.tw. OR exp Glasgow Outcome Scale/

64. Short-Form.tw. OR SF.tw.

65. patient-reported.tw. OR family-reported.tw. OR patient perspective.tw. OR family perspective.tw.

66. exp Social Adjustment/

67. readmission$.tw. OR exp Patient Readmission/

68. emergency department return$.tw. OR emergency room return$.tw.

69. multiple ED visit*.tw. OR multiple emergency department visit*.tw. OR multiple emergency room visit*.tw.

70. reintubation.tw.

71. unplanned ICU admission.tw. OR ICU readmission.tw. OR intensive care unit readmission.tw.

72. Reoperation*.tw. OR repeat* surger*.tw. OR exp Reoperation/

73. 14-72/or

74. 3 and 13 and 73

75. exp animals/ not humans.sh.

76. 74 not 75
